# The official position of the Latin American Association of Cardiac and Endovascular Surgery (LACES) regarding the recently released SOLACI/SIAC Clinical Guidelines on TAVI *versus* SAVR

**DOI:** 10.21470/1678-9741-2021-0385

**Published:** 2021

**Authors:** Victor Dayan, Ovidio A. Garcia-Villarreal, Alejandro Escobar, Javier Ferrari, Eduard Quintana, Mateo Marin-Cuartas, Rui M. S. Almeida

**Affiliations:** 1 Centro Cardiovascular Universitario, Montevideo, Uruguay.; 2 Mexican College of Cardiovascular and Thoracic Surgery, Mexico City, Mexico.; 3 Universidad CES, Medellin, Colombia.; 4 Colegio Argentino de Cirujanos Cardiovasculares, Buenos Aires, Argentina.; 5 Cardiovascular Surgery Department, Hospital Clinic Barcelona, Barcelona, Spain.; 6 University Department of Cardiac Surgery, Leipzig Heart Center, Leipzig, Germany.; 7 Department of Cardiothoracic Surgery, Stanford University, Stanford, United States of America.; 8 University Center Assis Gurgacz Foundation, Cascavel, Paraná, Brazil.

The Heart journal, from the British Medical Journal and the British Cardiovascular Society, has recently published a review titled “Clinical practice guideline for transcatheter (TAVI) versus surgical aortic valve replacement (SAVR) in patients with severe aortic stenosis in Latin America”^(1)^. Based on a weak (or conditional) recommendation, the panel concludes that “In elderly (75 years or older) patients living in Latin America with severe symptomatic aortic stenosis (AS) candidates for transfemoral approach, the panel suggests the use of TAVI over SAVR”. This guideline has been put together and funded by the Sociedad Latinoamericana de Cardiologia Intervencionista (SOLACI) with the endorsement of the Sociedad Interamericana de Cardiologia (SIAC). As stated by the authors, the rationale for this document emerges as a response to their disagreement with the decision made by the cost-effectiveness technology assessment agency in Argentina, which defined the use of transcatheter aortic valve replacement for inoperable patients. The aim was “to develop a high-quality and transparent guideline to help physicians and other stakeholders concerning the use of TAVI versus SAVR in Latin America”. Despite the authors’ thorough job reviewing the existing international evidence according to the Grading of Recommendations, Assessment, Development and Evaluations (GRADE) criteria and the relevant systematic review, these recommendations, which stem from Latin-American authors, are not specifically for Latin America.

The Latin American Association of Cardiac and Endovascular Surgery (LACES) nucleates over 400 members of the Latin-American continent. It started in 2019 and is currently the most inclusive cardiac surgical association in Latin America, recognized by the European Association of CardioThoracic Surgery, Society of Thoracic Surgeons, American Association of Thoracic Surgery, and Association of Thoracic and Cardiovascular Surgeons of Asia. Based on the following issues, this association does not support the SOLACI/SIAC recommendations.

A guideline to help physicians and stakeholders decide the best course of action in Latin America must come from Latin-American effectiveness data. It is impossible to recommend something for our region when we do not know what is going on. The effectiveness of a procedure depends not only on the procedure, per se, but also on the socioeconomic reality, which directly affects our patients and is the basis of the cost-effectiveness analysis. Several reports have highlighted the importance of hospital and technical volume to assure good outcomes^(2)^. Extrapolating data from a different socioeconomic reality with different hospital volume experiences to our continent is dangerous for our patients and the health economic system. When the aim is to perform regional guidelines, as in the title of this review, it is critical to consider the regional context; if not, this review may well apply to Africa, Europe, America, Asia, or Oceania. The authors explicitly disregarded Latin-American evidence under the assumption that the conclusions were “erratic”. Not including regional evidence is a significant flaw and suggests serious selection bias in the evidence process.

To begin with, the document fails to examine critical and recent evidence and make recommendations in line with the goals of treating heart valve disease. The treatment of AS should be accomplished aiming to restore long-term life expectancy and improve quality of life, avoiding detrimental late events reversing the early success of the procedure. For now, only SAVR is established to restore the prognosis of patients with symptomatic severe AS, with long-term postoperative survival becoming comparable to an age- and sex-matched general population without AS in patients over 65 years old^(3)^-^(5)^.

The SOLACI/SIAC document implicitly assumes an equipoise between long-term outcomes of TAVI and SAVR, which has been challenged by recent evidence. In this way, the results of randomized controlled trials, metanalyses, and national databases show the opposite^(6)^. The report of the five-year outcomes of the Placement of Aortic Transcatheter Valve (PARTNER) 2 cohort A trial found a higher risk of death or disabling stroke between two and five years after TAVI than after SAVR, with a hazard 27% higher^(7)^,^(8)^. The meta-analysis by Barili et al. with Kaplan-Meier-estimated individual patient data evaluating the effects of TAVI and SAVR on the long-term all-cause mortality rate revealed a lower incidence of death in the first year after TAVI. In contrast, there was a reversal of the endpoint after 40 months favoring SAVR over TAVI. The mortality rates in trials of TAVI *vs*. SAVR are affected by treatments with a time-varying effect and TAVI is related to better survival in the first months after implantation whereas, after 40 months, it is a risk factor for all-cause mortality^(9)^.

In the PARTNER 3 trial, the event-rate lines for death and disabling strokes, which significantly favored TAVI in the one-year analysis, from the two-year follow-up, the curves are converging over time, and reversal of fortunes may become tangible in the longer-term^(10)^.

The German Aortic Valve Registry (GARY), assessing the long-term outcomes of aortic valve replacement with TAVI and SAVR, revealed that after propensity score matching, TAVI with early generation prosthesis was associated with significantly higher five-year all-cause mortality than SAVR, reaffirming earlier data from Italian and French nationwide registries. Additionally, the curves for survival probability keep diverging over time, hinting at a longer-term worse prognosis for patients who underwent TAVI procedure. The finding from the GARY registry is worrisome as it raises the further question of what could be done to low- and intermediate-risk patients who had TAVI implanted and now may face shorter life expectancy^(11)^.

The recommendation for TAVI in patients over 75 years old is unrealistic, given that the life expectancy for men and women aged 75 is a further 11.18 and 12.97 years, respectively, therefore the long-term benefit of the procedure would be adversely affected cutting short late survival^(12)^.

During all these years, different centers from Latin America have generated essential data to produce high-quality effectiveness results and therefore adapt recommendations to our reality. We consider this is critical to produce trustworthy recommendations and inform stakeholders on the best action to take. Our association has always been open to working in a multidisciplinary team objectively to produce and analyze these data. We believe this is the initial step before making any regional guideline.

Severe conflicts of interest (COI) generate undisputed bias. Despite the fact that surgeons have proficiently been treating aortic valve stenosis for more than 60 years, no cardiovascular surgical association was invited to take part in this document. The authors mention that no surgical association exists in Latin America, which shows an apparent disrespect to LACES and the concept of Heart Team.

In fact, surgical cardiovascular societies have been recurrently excluded from making TAVI guidelines, raising the suspicion of primary stealth financial interest behind this^(13)^.

Citing Gordon Guyatt from GRADE, “We believe that the key to developing conflict-free recommendations is that panel members without conflicts and, in particular, the methodologist chapter editor, bear responsibility for the final presentation of evidence summaries and rating of the quality of evidence. The chapter editor is also responsible for ensuring that, during the discussion of evidence, panel members with conflicts do not take an aggressive advocacy role”^(14)^. The methodologist and principal investigator, Dr. Lamelas, has heavy COI with Edwards, Medtronic, and Boston Scientific. The voting panel included four members who are proctors for these companies as well. The methodology team, led by Dr. Lamelas, decided that “TAVI proctoring may not be a strong financial conflict of interest”. We strongly disagree with this decision and believe that Latin America has surgeons and interventional cardiologists with robust academic experience and without COI who are not proctors for the industry with the capacity to evaluate the evidence and provide an objective vote.

The authors mention that they did not involve SOLACI authorities in the process of the guidelines. This assertion is unclear since the principal investigator (PL) is the coordinator of research in SOLACI, and one of the external reviewers, Oscar Mendiz (for whom the COI are not disclosed), is part of the counseling committee of SOLACI.

The authors have mentioned but not analyzed the role that costs have on TAVI implementation in Latin America. They propose to waive taxes on TAVI as a solution for this limitation. Considering that the GRADE approach requires a multidisciplinary team with specific skills, it would have been critical to have experts from healthcare and health technology assessment from Latin America among the guideline team.

National per capita expenditures on health vary widely in Latin-American countries, with a mean of USD PPP 1,025, ranging from less than USD PPP 500 in some countries of Central America and the Andean region (Bolivia, Honduras, Guatemala, and Nicaragua), to around USD PPP 1,138 in Mexico, USD PPP 1,280 in Brazil, USD PPP 1,907 in Argentina, and USD PPP 2,484 in Cuba. These figures are in sharp contrast with the average Organisation for Economic Co-operation and Development (OECD) countries and United States of America 2019 health spending per capita of USD PPP 4,223 and USD PPP 11,071, respectively^(15)^.

In times of extreme pressure on health resources, regardless of the countries’ economic position, adopting a new technology that is five to 10 times more expensive than the existing standard, with inferior results, seems illogical and requires serious reflection^(16)^.

In summary, despite the extensive review of the current evidence of TAVI and SAVR, we believe the authors failed to examine the existing evidence from Latin America, comprehensively. Heavy COI in members of the voting panel and from the chair of the guideline (PL) make the process and recommendations unrealistic and biased. We believe guidelines should meet three essential requisites:

- Be based on the reality of the region where it intends to be applied
- Be led, as GRADE suggests, by an independent group of investigators
- Following GRADE recommendations, including experts from all critical disciplines

Furthermore, accurate and trustful information to patients and their family should be made available - the risks involved, the benefits afforded, and the expected long-term prognosis.

The extensive financial COI may unduly influence professional judgments, interfere with the appraisal and recommendation of the Heart Valve Team, and discredit the integrity of science, the quality of care, and public confidence in medicine. There is a debate that surgical and medical societies should compulsorily embrace and coordinate their efforts regarding the response to flawed trials and unrealistic guidelines.

LACES is open to working jointly for this and future guidelines under GRADE methodology and led an independent GRADE expert group. The stand taken by the Heart journal publishing the pretense SOLACI/SIAC guidelines with all the raised biases is troublesome. This document has been submitted as a Letter to the Editor to the Heart journal in response to its publication and, since no response was received after repeated e-mail contacts to the editorial office, a decision was made to submit it to the Brazilian Journal of Cardiovascular Surgery.
